# Safety and immunogenicity of novel respiratory syncytial virus (RSV) vaccines based on the RSV viral proteins F, N and M2-1 encoded by simian adenovirus (PanAd3-RSV) and MVA (MVA-RSV); protocol for an open-label, dose-escalation, single-centre, phase 1 clinical trial in healthy adults

**DOI:** 10.1136/bmjopen-2015-008748

**Published:** 2015-10-28

**Authors:** C A Green, E Scarselli, M Voysey, S Capone, A Vitelli, A Nicosia, R Cortese, A J Thompson, C S Sande, Catherine de Lara, P Klenerman, A J Pollard

**Affiliations:** 1 Oxford Vaccine Group, Department of Paediatrics, The NIHR Oxford Biomedical Research Centre, University of Oxford, Oxford, UK; 2 ReiThera Srl, (formerly Okairos Srl), Rome, Italy; 3 Nuffield Department of Primary Care Health Sciences, University of Oxford, Oxford, UK; 4 Experimental Medicine Division, Nuffield Department of Medicine, University of Oxford, Oxford, UK

**Keywords:** IMMUNOLOGY

## Abstract

**Introduction:**

Respiratory syncytial virus (RSV) infection causes respiratory disease throughout life, with infants and the elderly at risk of severe disease and death. RSV001 is a phase 1 (first-in-man), open-label, dose-escalation, clinical trial of novel genetic viral-vectored vaccine candidates PanAd3-RSV and modified vaccinia virus Ankara (MVA)-RSV. The objective of RSV001 is to characterise the (primary objective) safety and (secondary objective) immunogenicity of these vaccines in healthy younger and older adults.

**Methods and analysis:**

Heterologous and homologous ‘prime’/boost combinations of PanAd3-RSV and single-dose MVA-RSV are evaluated in healthy adults. 40 healthy adults aged 18–50 years test one of four combinations of intramuscular (IM) or intranasal (IN) PanAd3-RSV prime and IM PanAd3 or IM MVA-RSV boost vaccination, starting at a low dose for safety. The following year an additional 30 healthy adults aged 60–75 years test either a single dose of IM MVA-RSV, one of three combinations of IN or IM PanAd3-RSV prime and PanAd3-RSV or MVA-RSV boost vaccination used in younger volunteers, and a non-vaccinated control group. Study participants are self-selected volunteers who satisfy the eligibility criteria and are assigned to study groups by sequential allocation. Safety assessment includes the daily recording of solicited and unsolicited adverse events for 1 week after vaccination, as well as visit (nursing) observations and safety bloods obtained at all scheduled attendances. Laboratory measures of RSV-specific humoral and cellular immune responses after vaccination will address the secondary end points. All study procedures are performed at the Centre for Clinical Vaccinology and Tropical Medicine (CCVTM), Oxford, UK.

**Ethics and dissemination:**

RSV001 has clinical trial authorisation from the Medicines and Healthcare Products Regulatory Agency (MHRA) and ethics approval from NRES Berkshire (reference 13/SC/0023). All study procedures adhere to International Conference on Harmonisation (ICH) Good Clinical Practice guidelines. The results of the trial are to be published in peer-reviewed journals, conferences and academic forums.

**Trial registration number:**

NCT01805921.

## Introduction

Respiratory syncytial virus (RSV), is a major global health problem. RSV causes respiratory infections throughout life, with infants in the first months of life, severely immune-compromised adults and the elderly especially susceptible to developing severe lower respiratory tract disease or death.

Primary RSV infection is common in the first year of life and usually mild.^1^ Severe disease, manifesting as a compromise of breathing or feeding, warrants hospital admission in 2–3% of primary infections where the care is supportive until the immune response controls the infection to restore normal function.^2^ ^3^ Passive immune prophylaxis with monoclonal antibody can reduce hospitalisation rates in infants with predisposing conditions for developing severe disease, but the majority of RSV-bronchiolitis admissions are from otherwise healthy infants born at term.^4^ ^5^ Severe bronchiolitis accounts for 10% all annual admissions to paediatric intensive care units in the UK^3^ ^6^ and access to mechanical ventilation has maintained low mortality rates from RSV disease in industrialised healthcare settings.^7^ ^8^ Globally, RSV infection is the second most common cause of death in infants after 1 month and before 1 year of age^9^ and children under 5 years of age are responsible for an estimated 34 million lower respiratory tract infections, 3.4 million hospitalisations and up to 200 000 deaths worldwide each year.^10^ RSV infection in infancy has also been associated with the development of wheeze and possibly asthma later in later years, with accumulating evidence for causality.^11^ ^12^ Reinfection occurs throughout life and even possible within the first year of life.^13^ Healthy adults experience mild RSV infections^14^ ^15^ but severe immune-compromise can risk severe lower respiratory tract disease.^16^ An aging immune system and comorbidities in the elderly are factors thought to increase rates of hospitalisation and mortality from RSV to comparable estimates of disease burden to seasonal influenza.^17–20^

There is no licenced vaccine and RSV remains a high priority for vaccine development. The disastrous formalin-inactivated RSV (FI-RSV) vaccine trial in the in 1960s resulted in the death of two infants following natural exposure and a 16-fold rise in hospitalisation rate in other vaccine recipients.^21–24^ This tragedy, together with an incomplete understanding of the immune response and immune correlates of protection to natural disease, has made RSV vaccine development especially challenging. Concerns about vaccine primed immunopathology to natural exposure in RSV-naïve infants and inadequate immunogenicity in the elderly has focused RSV subunit vaccine development as maternal vaccines.^25–28^ Live-attenuated vaccine candidates have been in clinical evaluation for over 50 years and appear safe in seronegative infants, but remain troubled by genetic instability, limited immunogenicity and vaccine virus shedding with secondary infection.^29–32^

In this first-in-man clinical trial, designated RSV001, we characterise the safety and immunogenicity of novel viral-vectored vaccines used to deliver RSV antigen in healthy adult volunteers. Heterologous and homologous prime/boost combinations of genetically modified chimpanzee derived adenovirus and modified vaccinia virus Ankara (MVA) viral vectors have been used as biological platforms in the safe delivery of vaccine antigen for several infectious diseases and cancer, including safe use in infants.^33–36^ In RSV001, the replication-defective adenoviral vector PanAd3 and MVA encode 3 RSV proteins as antigen for the induction of humoral and cellular RSV-specific immune responses.

## Materials and analysis

### Objectives and end points of the clinical trial

The objectives of the RSV001 trial are to characterise the safety and tolerability (primary objective) and immunogenicity (secondary objective) of different combinations of PanAd3-RSV and MVA-RSV in healthy adults aged 18–50 and 60–75 years. The primary and secondary end points are listed in table 1.

**Table 1:** The primary and secondary objectives and end points to RSV001

		End point measures
Primary objective: vaccine safety and tolerability	Solicited and unsolicited symptoms recorded daily for 1 week after each vaccine dose	Oral temperature Solicited symptoms: headache, nausea and/or vomiting, malaise, myalgia and arthralgia Local injection site adverse events: pain and/or tenderness, erythema, induration and swelling Local nasal site adverse events: pain and/or tenderness, irritation and discharge Any other event not listed above (unsolicited symptoms)
	Nursing observations obtained at all visit attendances	Resting heart rate, resting respiratory rate, systolic and diastolic blood pressure and oral temperature
	Safety bloods obtained at scheduled visit attendances	Haematology: haemoglobin, total white cell count, platelet count, haematocrit, red cell count, mean cell volume, mean haemoglobin, mean haemoglobin concentration, neutrophil count, lymphocyte count, monocyte count, eosinophil count and basophil count Biochemistry: sodium, potassium, urea, amylase, C reactive protein, creatinine, bilirubin, alanine transaminase, alkaline phosphatase and albumin
Secondary objective: vaccine immunogenicity	Antibody response to vaccination	Serum antibody response to RSV F antigen Serum antibody response capable of RSV neutralisation
	Cellular immune response to vaccination	Quantification of circulating vaccine-induced antibody secreting B-cells (IgA and IgG) against RSV F antigen Quantification of circulating vaccine-induced T-cell responses against RSV antigens F, N and M2–1
	Exploratory immunology	Nasal and salivary antibody response to vaccination CD4+ and CD8+ T-cell subset response to vaccination Cytokine response to vaccination Gene expression changes after vaccination Any further exploratory immunology to detect vaccine-related immune responses

RSV, respiratory syncytial virus.

### Intervention

The generation of the PanAd3 vector and PanAd3-RSV and MVA-RSV vaccines, and results of preclinical evaluation, are described in more detail elsewhere.^37–40^

#### Vaccine construct

**Figure 1:**
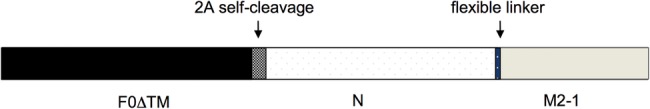
The single DNA fragment insertion for the expression of RSV proteins by PanAd3 and MVA. The same codon-optimised DNA fragment was inserted into replication-defective PanAd3 and MVA viral vectors. After transfection into a mammalian cell, cleavage of a Foot and Mouth Disease Virus 2A region releases a soluble truncated F protein while the N and M2-1 proteins remain intracellular. MVA, modified vaccinia virus Ankara; RSV, respiratory syncytial virus.

PanAd3-RSV and MVA-RSV are replication-defective genetically modified organisms (GMOs) for the delivery of RSV protein as vaccine antigen. The genetic insert for PanAd3 and MVA viral vectors is the same codon-optimised single synthetic DNA fragment encoding the RSV proteins F (fusion protein, F0ΔTM), N (nucleocapsid protein) and M2–1 (matrix protein), shown schematically in figure 1. Deletion of the E1 and E4 loci of PanAd3 renders the adenovirus replication defective, and MVA cannot naturally replicate in mammalian cells. The F0ΔTM protein is a truncated soluble antigen (lacking the trans-membrane region of the full length F protein) detectable in the cell supernatant while the N and M2–1 proteins are retained intracellularly after transfection of the vaccine virus. Product safety and characterisation testing included sterility, endotoxins, residual host cell DNA and proteins, genome sequencing and extensive screening for extraneous virus contamination. For PanAd3, the absence of replication-competent adenovirus was verified and potency of the product quantified using antihexon immune staining and quantitation of total vector particle concentration. Testing of the MVA-RSV vaccine product included virus titre, identity, purity and expression of RSV transgene. Clinical grade PanAd3-RSV and MVA-RSV vaccine products were manufactured under Good Manufacturing Practice (GMP) conditions by Advent (Italy) and Impfstoffwerk Dessau-Tornau (Germany). Vaccines are stored on site at ≤−60°C until use.

#### Preclinical data from PanAd3-RSV and MVA-RSV

Cotton rats, mice and calves were used to generate preclinical safety and immunogenicity data from PanAd3-RSV and MVA-RSV.^37–39^ These included human and bovine RSV challenge experiments in cotton rats and calves respectively, and included non-vaccinated and FI-RSV vaccinated controls. Seronegative calves were used as a translational model for seronegative infants who are at the greatest risk of developing severe RSV disease and FI-RSV associated immunopathology following natural exposure. In these experiments we could not detect any FI-RSV Th2-associated immunopathology after challenge. All combinations of vaccine were capable of protection from viral replication in the lower respiratory tract, and heterologous combinations of vaccine (including intra-nasal PanAd3-RSV prime) conferred complete restriction of viral replication in the upper respiratory tract as well. Based on data from the bovine challenge model,^37^ four heterologous and homolgous prime/boost combinations were selected for the RSV001 trial in humans.

### Clinical trial design

RSV001 is an open label, dose-escalation, single-site, phase 1 (first-in-man) clinical trial of PanAd3-RSV and MVA-RSV in healthy adult volunteers. 40 healthy volunteers aged 18–50 years are enrolled in 2013. After an interim analysis of safety and immunogenicity data, the following year an additional 30 healthy adults aged 60–75 years are required to assess safety and immunogenicity in older adults, as development of the vaccine towards a population in need of protection to severe RSV disease.

#### Setting and current progress

RSV001 is being performed at the Centre for Clinical Vaccinology and Tropical Medicine (CCVTM), Oxford, UK. Clinical procedures started in May 2013 and clinical data collection is expected to complete in August 2015. Laboratory analyses are expected to continue for a period up to 3 years from this date.

#### Study groups

RSV001 contains a total of nine study groups; seven groups are either heterologous or homologous prime/boost combinations of PanAd3-RSV and MVA-RSV, one group is single-dose MVA-RSV and one group is a non-vaccinated control arm (see table 2). Adults have robust RSV-specific immune responses from repeated exposure and the term ‘prime’ is inherited from clinical trials in antigen naïve populations. Here the term ‘prime’ is used to denote the first dose of vaccine, and ‘boost’ the second dose of vaccine.

**Table 2:** The nine study groups in RSV001

	N volunteers	Prime vaccine	Boost vaccine	Prime/boost interval
2013/14—healthy adults aged 18–50 years				
Group 1	10	IM PanAd3-RSV	IM MVA-RSV	8 weeks
Group 2	10	IM PanAd3-RSV	IM PanAd3-RSV	4 weeks
Group 3	10	IN PanAd3-RSV	IM MVA-RSV	8 weeks
Group 4	10	IN PanAd3-RSV	IM PanAd3-RSV	8 weeks
2014/15—healthy adults aged 60–75 years				
Group 5	6	None	None	Not applicable
Group 6	6	IM MVA-RSV	None	Not applicable
Group 7	6	IM PanAd3-RSV	IM PanAd3-RSV	4 weeks
Group 8	6	IN PanAd3-RSV	IM MVA-RSV	8 weeks
Group 9	6	IM PanAd3-RSV	IM MVA-RSV	8 weeks

PanAd3-RSV prime can be administered by IM injection or IN spray. All boost vaccines are given by IM injection. The first two volunteers in groups 1–4 receive a low-dose of vaccine; all other volunteers in RSV001 receive the high-dose of vaccine. The prime/boost interval is 8 weeks for all study groups who receive two doses of vaccine except for the double prime groups 2 and 7 who are vaccinated with a 4-week interval between doses.

IM, intramuscular; IN, intranasal; MVA, modified vaccinia virus Ankara; RSV, respiratory syncytial virus.

Adults aged 18–50 years test one of four combinations of prime/boost (study groups 1–4) selected from preclinical data in animal challenge experiments, and include an intranasal route for the adenoviral vectored prime dose. Within each study group, two individuals test a low-dose vaccine before the remaining volunteers receive high-dose vaccine.

Adults aged 60–75 years have a non-vaccinated control group, a single high-dose MVA-RSV group and three groups testing different combinations of prime/boost vaccine at high dose (study groups 5–9). The vaccine strategies for older adults are selected from the interim analysis of safety and immunogenicity data from study groups 1–4 in younger adults.

#### Dose escalation

**Figure 2:**
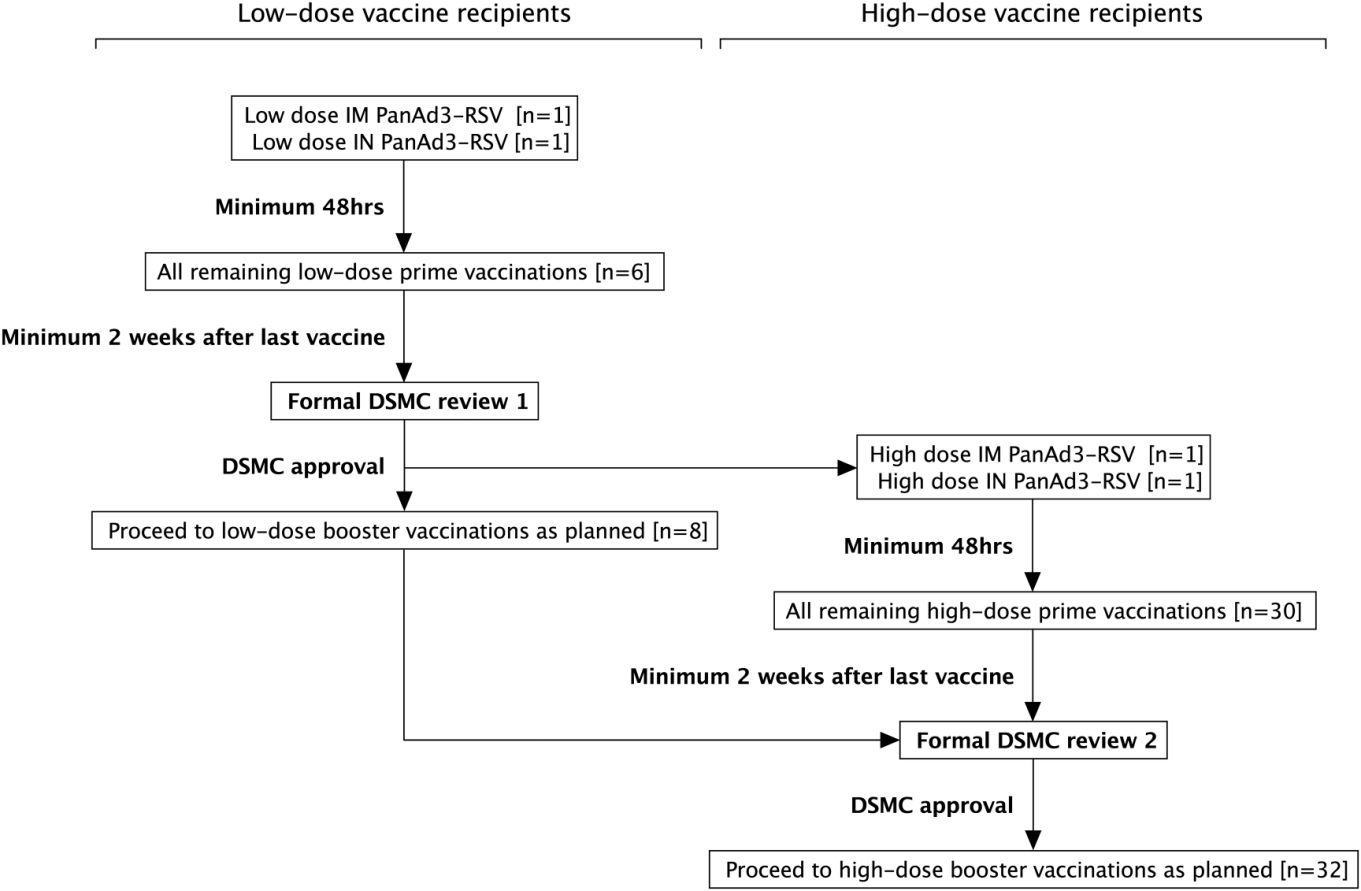
Schematic for dose and group size escalation for adults aged 18–50 years, enrolled in 2013. (n=number of volunteers). At each dose, prime vaccination with IM and IN PanAd3-RSV was performed in one individual and safety assessed for a minimum of 48 h before the remaining prime vaccinations were administered. Formal DSMC approval was required before administration of the low-dose booster and high-dose prime vaccinations, and again for the high-dose boost vaccinations. For older adults aged 60–75 years, enrolled in 2014, there is no low dose of vaccine and no group size escalation. DSMC, data safety monitoring committee; IM, intramuscular; IN, intranasal; RSV, respiratory syncytial virus.

The trial commences with the low-dose vaccinations in younger adults. One volunteer receives IM PanAd3-RSV and one volunteer IN PanAd3-RSV and are observed for any adverse events (AEs) for a minimum of 48 h before any more volunteers receive low-dose prime vaccine. Formal assessment of all safety data and approval from the independent data safety monitoring committee (DSMC) is required before dose escalation and continued (booster) low-dose vaccinations. Halting rules for dose-escalation includes the occurrence of a serious adverse reaction (SAR) or if two participants experience a severe AE (grade 3) that is clinically significant with a reasonable possibility of being related to the vaccine. If the trial is halted, study drug is not administered to any additional subject and enrolment stops until the DSMC has reviewed the events. Dose and group size escalation in RSV001 are shown schematically in figure 2. All vaccines administered to groups 6–9 are at high dose.

#### Vaccination procedures

Doses of vaccine are prepared by diluting the concentrated product with 0.9% sterile saline solution to the required concentration and volume. Low-dose and high-dose PanAd3-RSV is 5×10^9^ and 5×10^10^ viral particles (vp), respectively. Low-dose and high-dose MVA-RSV is 1×10^7^ and 1×10^8^ plaque forming units (pfu), respectively. PanAd3-RSV is given either by intramuscular injection of 0.5 mLs vaccine product to the non-dominant deltoid muscle, or by intra-nasal spray of 0.15 mLs volume to each nostril in the sitting position using a syringe attached to an LMA MAD Nasal needle-free drug delivery system (LMA, San Diego, California, USA). MVA-RSV is administered by intra-muscular (IM) injection of 0.5 mLs vaccine product to the non-dominant deltoid muscle only.

Volunteers are required to wait and be observed for 1 h after the administration of each vaccine dose for signs of anaphylaxis. All clinical staff are trained and can provide evidence of competency in the acute management of anaphylaxis reactions and epinephrine is available at all times of vaccine administration and subsequent observation. This is detailed in relevant standard operating procedures (SOPs). The nearest medical ward is in the adjoining corridor and nearest Accident and Emergency Department is at the Oxford Radcliffe Hospital, Oxford University Hospitals NHS Trust, which is within minutes by ambulance transfer.

#### GMO considerations

PanAd3-RSV and MVA-RSV are used in accordance with GMO (Contained Use) Regulations 2000. Vaccine administered by intramuscular injection has the site of inoculation covered with a dressing after immunisation. This should absorb any virus that may leak out through the needle track and the dressing is removed from the injection site after 30 min and disposed as GMO waste by autoclaving in accordance with relevant SOPs. Each volunteer vaccinated with PanAd3-RSV by intranasal spray remains in the clinic room for 30 min after dosing to contain any vaccine expelled by sneezing. The Mucosal Atomization Device (MAD nasal) is disposed as GMO waste by autoclaving in accordance with the relevant SOPs.

#### Study procedures and schedules for data collection

A volunteer is considered enrolled at the point of receiving the first vaccine (prime). For Group five volunteers, who do not receive a vaccine, enrolment occurs with completion of the first visit (V1). If a volunteer withdraws from the study, a newly recruited volunteer may commence the trial in their place. The schedule of research site attendances, study procedures and data collection for enrolled volunteers are set out in table 3. In summary;

- Volunteers in study groups 1, 3 and 4 are invited to attend a total of 12 visits over 34 weeks
- Volunteers in study group 2 are invited to attend a total of 11 visits over 30 weeks
- Volunteers in study groups 5, 8 and 9 are invited to attend 13 visits over 52 weeks
- Volunteers in study groups 6 and 7 are invited to attend 12 visits over 52 weeks

**Table 3:** Table of procedures for RSV001

	Days after vaccination (±window, days)	Vaccine continued eligibility	Solicited adverse events	Unsolicited adverse events	Visit observations	Nasal sample for RSV detection	Nasal sample for PanAd3-RSV detection*	Nasal sample for anti-F antibody	Salivary sample for anti-F antibody	Safety bloods (mLs)	Serum anti-PanAd3 antibody (blood, mLs)	Serum anti-F antibody (blood, mLs)	B-cell responses (blood, mLs)	T-cell responses (blood, mLs)	RNA for gene expression analysis† (blood, mLs)
V1 (P)	NA	♦	♦	♦	♦			♦	♦	5 5	5 5	10 7	30 25	60 25	3 3
V2	3±1		♦	♦	♦		♦			5 5					3 3
V3	7±2		♦	♦	♦					5 5			30 25		3 3
V4	14±3			♦	♦			♦	♦	5 5		10 7		60 50	
V5	28±4			♦	♦			♦	♦	5 5		10 7		60 50	3 3
V6 (B)	NA	♦	♦	♦	♦			♦	♦	5 5	5 5	10 7	30 25	60 25	3 3
V7	3±1		♦	♦	♦					5 5					3 3
V8	7±2		♦	♦	♦					5 5			30 25	60 25	3 3
V9	14±3			♦	♦			♦	♦	5 5		10 7		60 50	
V10	28±4			♦	♦			♦	♦	5 5	5‡ 5‡	10 7	30 25	60 25	3 3
V11	70±7			♦	♦			♦	♦	5 5		10 7	30 25	80 25	
V12	182±12			♦	♦					5 5		10 7		60 50	
V13§	332±28			♦	♦					5 5		NA 7	NA 25	NA 25	
UN	Any			♦	♦	♦						10 7			

*Nasal samples for PanAd3-RSV shedding was performed only in volunteers who received IN PanAd3-RSV prime (groups 3, 4 and 8).

†No RNA samples were taken from the single-dose MVA group (group 6) from V6 onwards.

‡Serum anti-PanAd3 antibody samples at V10 were obtained from volunteers who received IM PanAd3-RSV vaccine at prime and boost (groups 2 and 7).

§There is no V13 time-point for adults aged 18–50 years (study groups 1–4). For each blood sample, the top number is the volume obtained from adults aged 18–50 years (study groups 1–4); the bottom number is the volume obtained from adults aged 60–75 years (study groups 5–9). In addition to scheduled attendances, each volunteer was allowed a maximum of four unscheduled visits (UN1, UN2, UN3 and UN4) for reasons of safety or influenza-like illnesses and the detection of RSV infection.

♦Denotes collection of these data at these visits. Each volunteer also attended a screening visit (V0) before enrolment into the study and receives prime vaccine (P) at V1 and boost vaccine (B) at V6. The prime/boost interval is group dependent. Study groups 2 and 7 do not have a V5 and group 6 does not have a V7. Vaccine continued eligibility tests include an interim medical history (see temporary exclusion criteria) and urine pregnancy testing.

IN, intranasal; MVA, modified vaccinia virus Ankara; NA, not applicable; RSV, respiratory syncytial virus.

Volunteers who report an influenza-like illness during the study attend an unscheduled visit for the detection of RSV or other viral infection by PCR on nasal swabs. This may identify RSV-specific immune responses to natural infection that could mimic vaccine immunogenicity and four unscheduled visits per volunteer are permitted.

The maximum volume of blood obtained from any volunteer in study groups 1–4 is 964 mLs from screening to completion of all subsequent visits inclusive of four unscheduled visits. In older adults (study groups 5–9) the maximum volume of blood obtained from any volunteer is 725 mLs from screening to completion of all subsequent visits inclusive of four unscheduled visits. DNA and/or serum remaining after the RSV001 analyses have been performed are, with specific volunteer consent, to be transferred to the Oxford Vaccine Group BioBank for future seroepidemiological and other vaccine related research (ethics reference LREC 10/H0504/25).

### Study volunteers

#### Recruitment

Potential volunteers are self-selected members of the public responding to advertisements inviting them to consider volunteering for the trial. Methods of recruitment include;

- Poster advertising throughout local hospitals and doctor's surgeries, tertiary education institutions and other public places with the permission of the owner/proprietor.
- Direct mail-out of the study information sheet to adults whose names and addresses are obtained via the Electoral register. Those people who have indicated they do not wish to receive postal mail−shots would have their names removed prior to the Investigators being given the names and addresses. The company providing this service is registered under the Data Protection Act 1998.
- Email campaign via representatives of local tertiary education establishments and local employers and ask them to circulate posters and link to study website by email or hard copy.
- Oxford Vaccine Centre (OVC) database for healthy volunteers: Direct email and link to members of the public who have registered their interest in potentially volunteering for clinical trials conducted by OVC. This secure database is maintained by OVC and members of the public registered here have given consent to have their details recorded and be contacted expressly for this purpose of being notified when a trial opens for recruitment. They understand this is not a commitment to volunteering for any trial they are contacted about.
- Media advertising by local media, newspaper and website advertisement placed in locations relevant for the target age group with brief details of the study and contact details for further information.
- Website advertising on the OVG website (http://www.ovg.ox.ac.uk).
- Exhibitions using stalls or stands at exhibitions and/or fairs, such as University Fresher's Fairs.
- Open Exeter: Potential study volunteers identified via National Health Applications and Infrastructure Services (NHAIS) who hold the central NHS patient database (Open Exeter).

Potential volunteers who are interested in study participation are able to contact the Oxford Vaccine Group by telephone, email and website for further information. If potential volunteers are willing to proceed they are invited for physician screening, consent and formal assessment of suitability for the trial.

#### Informed consent

Consent is obtained from a study physician and each volunteer personally signs and dates the latest approved version of the informed consent form before any study specific procedures are performed. Written and verbal versions of the participant information booklet and informed consent form are presented to volunteers, detailing no less than the exact nature of and the rationale for performing the study, the implications and constraints of the protocol and the risks and benefits involved in taking part. It is clearly stated that volunteers are free to withdraw from the study at any time, for any reason and that they are under no obligation to give the reason for withdrawal. Potential volunteers allowed an adequate time to consider the information from when they receive it, and the opportunity to question the researcher, their GP or other independent parties to decide whether to participate in the study.

#### Assessment of eligibility

Formal assessment of trial eligibility follows once written informed consent is obtained. The full inclusion and exclusion criteria are detailed in box 1. Physician screening includes;

- Recording of volunteer demographics; age, sex, body mass index, ethnicity, occupation
- Assessment of current and past medical history
- Physical examination
- 12-lead ECG
- Blood sampling; routine haematology and biochemistry, blood born viruses (HIV, HBV and HCV) and serum IgA levels
- Urine sampling; microscopic haematuria, pyuria or proteinuria, and pregnancy if appropriate
- Registration with The Over-volunteering Prevention Systems (TOPS) to guard against the potential for harm that can result from excessive volunteering in clinical trials and blood donations
- GP agreement that they do not know of a reason the potential volunteer should be excluded, based on study specific paperwork sent to the GP.

An eligible or enrolled volunteer can have vaccination deferred if any of the following temporary exclusion criteria apply;

- Low-grade febrile illness (oral temperature ≥37.5°C)
- Any moderate or severe illness irrespective of temperature, such as diarrhoea, mild upper respiratory infection
- A laboratory AE considered, in the opinion of the investigator, of requiring of further time and/or investigation to resolve or stabilise prior to another dose of vaccine being administered
- Receipt of a live vaccine within 4 weeks prior to vaccination or a killed vaccine within 7 days prior to vaccination.

Box 1 **Inclusion and exclusion criteria for RSV001 volunteers**

Inclusion criteria—all must be satisfied

1. Volunteer must be willing and able to consent to take part in the clinical trial
2. At the time of enrolment; aged 18–50 years inclusive for study groups 1–4, or aged 60–75 years inclusive for study group 5–9
3. In good health as determine by medical history, physical examination and in the judgement of the study investigators
4. Willing to use effective contraception (if sexually active);
  - Females: Oral contraceptive pill, contraceptive implant or barrier methods from 1 month prior and for the duration of the study
  - Males: Barrier contraceptive from V1 until 3 months after the last dose of vaccine
5. Willing to allow his/her General Practitioner and/or Consultant, if appropriate, to be notified of participation in the study
6. Confirmation from the General Practitioner that they are aware of the inclusion and exclusion criteria and are satisfied from their knowledge of the volunteer that they are suitable to enrol
7. Willing to provide their National Insurance/Passport number for the purpose of TOPS registration

Exclusion criteria—none can be satisfied

1. History of any significant organ/system disease that interfere with trial conduct or completion. These include any history of significant disease in the following;
  - Cardiovascular disease including congenital heart disease, previous myocardial infarction, valvular heart surgery (or history of rheumatic fever), previous bacterial endocarditis, history of cardiac surgery (including pacemaker insertion), personal or family history of cardiomyopathy or sudden death
  - Respiratory disease such as asthma (excluding childhood asthma not treated in adulthood) and chronic obstructive pulmonary disease
  - Endocrine disorders such as diabetes mellitus and Addison's disease
  - Significant renal or bladder disease, including a history of renal calculi
  - Biliary tract disease
  - Gastro-intestinal disease such as inflammatory bowel disease, abdominal surgery within the last 2 years, coeliac disease and liver disease
  - Neurological disease such as seizures and myasthaenia gravis
  - Metabolic disease such as glucose-6-phosphate dehydrogenase deficiency
  - Psychiatric illness requiring hospitalisation or depression whose severity is deemed clinically significant by the chief investigator, consultant or GP
  - Non-benign cancer, including squamous cell carcinoma, basal cell carcinoma of the skin and cervical carcinoma in situ
  - Clinically significant contact dermatitis
2. Have any known or suspected impairment or alteration of immune function, resulting from, for example: congenital or acquired immune deficiency, HIV or symptoms/signs of an HIV-associated condition, autoimmune disease, receipt of immunosuppressive therapy such as chemotherapy or radiation therapy in the preceding 12 months or long-term corticosteroid therapy, receipt of immunoglobulin or any blood product transfusion within 3 months of the study start.
3. A vaccination history indicative of; planning to receive another vaccine (other than the study vaccine) within 4 weeks of vaccination, a history of anaphylaxis reaction to a vaccine, history of allergic disease or reactions likely to be exacerbated by any component of the vaccine (eg, Kathon), previously having received a recombinant simian or human adenoviral vaccine, previously having received a recombinant MVA vaccine.
4. Detection of any of the following at screening; IgA deficiency, anti-HIV antibody, hepatitis B surface antigen, anti-HCV antibody, any other significant abnormalities at the discretion of the study investigator.
5. Known or suspected drug and/or alcohol misuse (alcohol misuse defined as an intake >42 units/week).
6. Nasal septal pathology including; congenital deformities such as an abnormal septum or polyps, previous cauterisation, rhinoplasty or surgery of any kind, recurrent epistaxis.
7. Scheduled procedures requiring general anaesthesia during the study.
8. Participation in another research study involving an investigational product in the past 12 weeks, or are planning to do so during the study.
9. Inability, in the opinion of the investigator, to comply with study requirements.
10. Female volunteers who are pregnant, lactating or planning pregnancy during the course of the study.
11. Has donated blood within 4 months before starting the trial, or is intending to donate blood during the trial and up to 12 weeks after completing the trial.
12. Any other significant disease or disorder which, in the opinion of the investigator, may put the volunteer at risk because of participation in the study, influence the result of the study or impair the volunteer's ability to take part on the trial.

HCV, hepatitis C virus; GP, general practitioners; MVA, modified vaccinia virus Ankara; RSV, respiratory syncytial virus.

#### Allocation to study groups

Each volunteer is assigned to a study group by sequential allocation, and not by choice or by randomisation. Although RSV001 is not a randomised clinical trial, the results of volunteer recruitment, enrolment and retention are reported according to the CONSORT 2010 statement.^41^

#### Volunteer compensation

Enrolled volunteers are compensated for their time, costs incurred and the inconvenience of procedures based on the following figures:

- Travel expenses: £15 per visit
- Inconvenience of blood tests: £10 per blood donation
- Inconvenience of nasal sampling: £10 per sample
- Time required for visits: £20 per visit

Four extra visits (designated as unscheduled visits) are included in the total remuneration for each volunteer regardless of whether they are used to remove any incentive to report an influenza-like illness during the study. Volunteers in study group 2, 6 and 7 are compensated the same as all other volunteers despite having one less scheduled visit. Remuneration is on a *pro rata* basis should a volunteer fail to complete all scheduled visits and/or study requirements. Each volunteer in study groups 1–4 can receive a maximum of £845 and each volunteer in study groups 5–9 can receive a maximum of £920.

### Assessment of safety

#### Data collection and severity grading

All safety data are collected and analysed un-blinded onto an OpenClinica (Community edition) database. Volunteers report daily on the occurrence and severity of local and systemic solicited and unsolicited AEs for 1 week after vaccination. Severity grading is described in table 4. Visit observations (pulse, respiratory rate, temperature and blood pressure) measures from every visit are graded as described in table 5. Haematological and biochemical safety blood measures are obtained at all scheduled attendances and graded according to modified Federal Drug Administration (FDA) and Division of AIDS (DAIDS) criteria, set out in table 6. A nasal swab for the detection of vaccine virus shedding is performed 3 days after prime for volunteers in groups 3, 4 and 7 (IN PanAd3-RSV prime).

**Table 4:** Adverse event grading criteria for solicited and unsolicited events after vaccination

	Adverse event	Severity grade	Definition
Systemic adverse events	Oral temperature	0	<37.6**°**C
		1	37.6–38.0**°**C
		2	38.1–39.0**°**C
		3	>39.0**°**C
	Headache, nausea and/or vomiting, malaise, myalgia and arthralgia and any other systemic symptom	0	Absence or resolution of symptom
		1	Awareness of symptom but tolerated; transient or mild discomfort, little/no medical intervention required
		2	Discomfort enough to cause limitation of usual activity; some medical intervention or therapy required
		3	Incapacitating, absent from work or bed rest required; hospitalisation possible
Local adverse events after IM injection	Tenderness/pain at the site of injection	0	None
		1	Painful to touch; easily tolerated
		2	Painful when the limb is moved; interferes with daily activity
		3	Severe pain at rest; prevents daily activity
	Redness and induration at the site of injection	0	0–2 mm maximal diameter reaction
		1	3–50 mm maximal diameter reaction
		2	51–100 mm maximal diameter reaction
		3	>100 mm maximal diameter reaction
	Swelling at the site of injection	0	No visible reaction
		1	1–20 mm maximal diameter reaction
		2	21–50 mm maximal diameter reaction
		3	>50 mm maximal diameter reaction
Local adverse events after IN spray	Nasal tenderness/pain	0	None
		1	Painful on touch only and minimal
		2	Painful without touch, moderate severity
		3	Painful without touch, severe and interferes with smell
	Nasal irritation	0	None
		1	Mild irritation only
		2	Moderate irritation, distracting
		3	Severe irritation, disturbs rest or interferes with normal activity
	Nasal discharge	0	None
		1	Mild discharge
		2	Moderate discharge, readily controlled
		3	Severe discharge, interferes with normal activity

Adverse events are graded as 0 (normal), 1 (mild), 2 (moderate) or 3 (severe) from the recorded outcome measures in this table.

IM, intramuscular; IN, intranasal.

**Table 5:** Adverse event grading criteria for visit observations

	Adverse event	Severity grade	Definition
Systemic adverse events	Oral temperature (**°**C)	0	<37.6
		1	37.6–38.0
		2	38.1–39.0
		3	>39.0
Heart rate (beats/min)	Tachycardia	0	55–100
		1	101–115
		2	116–130
		3	>130
	Bradycardia	0	55–100
		1	50–54
		2	45–49
		3	<45
Blood pressure (mm Hg)	Systolic hypotension	0	90–140
		1	85–89
		2	80–84
		3	<80
	Systolic hypertension	0	90–140
		1	141–150
		2	151–155
		3	>155
	Diastolic hypertension	0	<91
		1	91–95
		2	96–100
		3	>100
Respiratory rate (breaths/min)	Tachypnoea	0	<17
		1	17–20
		2	21–25
		3	>25

Adverse events are graded as 0 (normal), 1 (mild), 2 (moderate) or 3 (severe) from the recorded outcome measures in this table.

**Table 6:** Adverse event grading criteria for safety bloods

	Grade 1 (Mild)	Grade 2 (Moderate)	Grade 3 (Severe)	Grade 4 (Emergency)
Haematology				
Haemoglobin change from baseline (g/dL)	<1.5	1.5–2.0	2.1–5.0	>5
Total white cell count (×10^9^/L): elevated	10.8–15.0	15.1–20.0	20.1–25.0	>25.0
Total white cell count (×10^9^/L): depressed	2.5–3.5	1.5–2.4	1.0–1.4	<1.0
Neutrophil count (×10^9^/L)	1.5–2.0	1.0–1.4	0.5–0.9	<0.5
Platelet count (×10^9^/L)	125–140	100–124	25–99	<25
Biochemistry				
Sodium (mmol/L): hyponatraemia	132–134	130–131	125–129	<125
Sodium (mmol/L): hypernatraemia	144–145	146–147	148–150	>150
Potassium (mmol/L): hypokalaemia	3.5–3.6	3.3–3.4	3.1–3.2	<3.1
Potassium (mmol/L): hyperkalaemia	5.1–5.2	5.3–5.4	5.5–5.6	>5.6
Urea (mmol/L)	8.2–8.9	9.0–11	>11	RRT
Creatinine (µmol/L)	132–150	151–176	177–221	>221 or RRT
ALT or AST (IU/L)	1.1–2.5×ULN	2.6–5.0×ULN	5.1–10×ULN	>10×ULN
Bilirubin (µmol/L): with increased ALT/AST	1.1–1.25×ULN	1.26–1.5×ULN	1.51–1.75×ULN	>1.75×ULN
Bilirubin (µmol/L): with normal ALT/AST	1.1–1.5×ULN	1.6–2.0×ULN	2.0–3.0×ULN	>3.0×ULN
Alkaline phosphatase (IU/L)	1.1–2.0×ULN	2.1–3.0×ULN	3.1–10×ULN	>10×ULN
Amylase (IU/L)	1.1–1.5×ULN	1.6–2.0×ULN	2.1–5.0×ULN	>5.0×ULN
Albumin (g/L): hypoalbuminaemia	28–31	25–27	<25	NA
C reactive protein (mg/L)	11–30	31–100	101–200	>200

ALT, alanine transaminase; AST, aspartate transaminase; NA, not applicable; RRT, renal replacement therapy; ULN, upper limit of normal.

#### Safety data monitoring

Continuous safety monitoring occurs throughout the trial by the study team with oversight from the DSMC. The DSMC is independent and reviews safety data throughout the study according to a prespecified DSMC charter. The DSMC charter is written in accordance with DAMOCLES guidance and agreed on before the trial starts.^42^ Formal approval from the DSMC is required prior to the administration of the low-dose boost vaccine and before dose escalation to high-dose prime, and again before high-dose boost (see figure 2). The outcome of each DSMC review is communicated directly to the study investigators and documentation of all reviews are kept in the trial master file. The Chair of the DSMC can also be contacted for advice where the chief investigator feels independent advice or review is required.

#### AE definitions and reporting

International Conference on Harmonisation (ICH) definitions are used for AEs, adverse reactions (ARs), severe AEs (SAEs), SARs and suspected unexpected SARs (SUSARs). A medically qualified individual must determine the relationship of each AE to the vaccine as either ‘related to the vaccine’ (reasonable temporal sequence and not reasonably attributed to another cause) or ‘not related to the vaccine’. Each AE should be recorded to represent a single diagnosis. Changes in laboratory values are only considered to be AEs if they are judged to be clinically significant, for example, if some action or intervention is required. It is left to the investigator's clinical judgment whether or not an AE is of sufficient severity to require the volunteer's removal from treatment. A volunteer may also voluntarily withdraw from treatment due to what he or she perceives as an intolerable AE. If either of these occurs, the volunteer must undergo an end of study assessment and be given appropriate care under medical supervision, by referral to their GP, until symptoms cease or the condition becomes resolved or is stable. All SAEs and SUSARs must be recorded and reported to the DMSC chair and sponsor within 24 h of discovery or notification of the event. Fatal or life-threatening SUSARs must be reported within 7 days and all other SUSARs within 15 days. Any additional relevant information should be sent within 8 days of the report.

#### Pregnancy

Although not AEs, pregnancies are reportable events. Should a volunteer become pregnant during the trial the vaccination is discontinued. Any pregnancy occurring during the clinical study and the outcome of the pregnancy is recorded and followed up for congenital abnormality or birth defect. Pregnancy notification and follow-up reports are provided to the DSMC Chair.

#### Trial monitoring

Clinical Trials Research Governance (CTRG), University of Oxford, will perform monitoring according to ICH Good Clinical Practice (GCP). Following written SOPs, the monitors verify that the clinical trial is conducted and data are generated, documented and reported in compliance with the protocol, GCP and the applicable regulatory requirements.

### Assessment of immunogenicity

All laboratory assays and data analyses are performed blinded. A summary of the planned assays is listed below and in tables 1 and 3.

- Serum antibody response to RSV F antigen
- Serum antibody response capable of RSV-neutralisation
- Quantification of circulating vaccine-induced antibody secreting B-cells (IgA and IgG) against RSV F antigen
- Quantification of circulating vaccine-induced T-cell responses against RSV antigens F, N and M2–1
- Any further exploratory immunology to detect vaccine-related immune responses.

### Statistics and analysis plan

Analyses are descriptive in nature; the purpose of the study is to characterise the safety and immunogenicity of PanAd3-RSV and MVA-RSV in healthy adults. There are no prespecified hypotheses on which to power the study. No formal sample size calculations are performed, as the sample sizes are standard to assess phase 1 stage product safety and tolerability.

Summary statistics are calculated for safety and immunogenicity end points without imputation for missing data. The Centre for Statistics in Medicine (CSM) writes a detailed statistical analysis plan before any data is examined. Interim study reports for study groups 1–4 and 5–9 are generated to examine safety and select immunogenicity data 1 month after the last dose of vaccine in each age group.

### Regulatory approvals and trial registration

Clinical Trials Authorisation is granted from the Medicines and Healthcare Products Regulatory Agency (MHRA, 07/01/2013) and University of Oxford Genetic Modification Safety Committee (GSMC reference GM462.11.64, 21/01/2013). Research Ethics Committee (REC) approval, and amendments, is granted (NRES Berkshire reference 13/SC/0023, 08/03/2013). Local site and NHS R&D approvals are granted (23/04/2013). RSV001 is registered with clinicaltrials.gov (ref NCT01805921) and EudraCT (ref 2011–003 589–34). The final protocol is V.6.1 (dated 24 September 2014).

## Ethics and dissemination

### Ethical and safety considerations

The Investigator ensures the study is conducted in accordance with the principles of the current version of the Declaration of Helsinki, and that RSV001 is conducted in full conformity with relevant regulations and with the current version of the ICH guidelines for GCP (CPMP/ICH/135/95). The trial staff ensure that the volunteer's anonymity is maintained. The study complies with the Data Protection Act, which requires data to be anonymised as soon as it is practical to do so, and all documents are stored securely and only accessible by trial staff and authorised personnel.

#### Potential risks and benefits of taking part

The risks of phlebotomy (pain and bruising) and common AEs after vaccination are explained to each potential volunteer before vaccination. The first-in-man nature of the PanAd3 adenoviral vector, used here as PanAd3-RSV, and the first MVA-RSV formulation draws on safety data derived from these vaccines in preclinical evaluation and from similar adenoviral-vectored and MVA-vectored vaccines used in humans against other infectious diseases. In particular, the absence of any enhanced respiratory disease (ERD) associated with FI-RSV immunopathology in animal challenge experiments and the independent assessment of preclinical data by the MHRA. Furthermore, all adults have had prior exposure to RSV and this was a major protective factor against developing ERD in the FI-RSV trial.^43^ ^44^ It is made clear that taking part in RSV001 cannot be assumed to have any benefit to study volunteers in protection to severe RSV infection. The only direct benefits are the investigation of their general health and altruistic satisfaction in supporting the clinical development of these vaccines.

#### Blood volumes

The maximal total blood volume taken over the total duration of the trial is within current National Health Service Blood and Transplant (NHSBT) allowances for a blood donation of 470 mLs every 12 weeks for males and every 16 weeks for females over the duration of the trial. As a further precaution, potential volunteers are excluded if donated blood within 4 months before entering the trial, or were planning to donate blood during the trial or within 12 weeks after the final visit. Haemoglobin measures are obtained at every visit and study investigators monitor changes in haemoglobin, with all data communicated to the DSMC for independent assessment. For adults aged 60–75 years, the total blood volume is reduced in response to the older age range and potential for slower recovery of haemoglobin due to phlebotomy.

#### Volunteer compensation

Volunteers are not paid to take part but are compensated for any cost and inconvenience with volunteering to take part in the trial. The tariff for each procedure, published here, affords accountability of the final compensation sum and allows for *pro rata* compensation for volunteers who do not complete all the study requirements. All four unscheduled visits are compensated, irrespective of whether they are needed, to remove any incentive to report an influenza-like illness during the trial. As volunteers cannot choose which study group to enter, but consent to join to any group, the omitted visits in some groups are compensated for in any case.

### Publication

The investigators have access to all data and co-ordinate the dissemination of data from the RSV001 clinical trial. All publications (eg, manuscripts, abstracts, oral/slide presentations, book chapters) based on this study are reviewed by each subinvestigator and by the sponsor prior to submission. Study volunteers are not given individual immunogenicity results but have the overall results of the clinical trial communicated to them directly by way of published material.
